# Adventitial Vasa Vasorum Arteriosclerosis in Abdominal Aortic Aneurysm

**DOI:** 10.1371/journal.pone.0057398

**Published:** 2013-02-27

**Authors:** Hiroki Tanaka, Nobuhiro Zaima, Takeshi Sasaki, Takahiro Hayasaka, Naoko Goto-Inoue, Kenji Onoue, Koji Ikegami, Yoshifumi Morita, Naoto Yamamoto, Yuuki Mano, Masaki Sano, Takaaki Saito, Kohji Sato, Hiroyuki Konno, Mitsutoshi Setou, Naoki Unno

**Affiliations:** 1 Second Department of Surgery, Hamamatsu University School of Medicine, Hamamatsu, Japan; 2 Department of Cell Biology and Anatomy, Hamamatsu University School of Medicine, Hamamatsu, Japan; 3 Department of Anatomy and Neuroscience, Hamamatsu University School of Medicine, Hamamatsu, Japan; 4 Department of Applied Biological Chemistry, Kinki University, Higashiosaka City, Japan; Brigham and Women’s Hospital, Harvard Medical School, United States of America

## Abstract

Abdominal aortic aneurysm (AAA) is a common disease among elderly individuals. However, the precise pathophysiology of AAA remains unknown. In AAA, an intraluminal thrombus prevents luminal perfusion of oxygen, allowing only the adventitial vaso vasorum (VV) to deliver oxygen and nutrients to the aortic wall. In this study, we examined changes in the adventitial VV wall in AAA to clarify the histopathological mechanisms underlying AAA. We found marked intimal hyperplasia of the adventitial VV in the AAA sac; further, immunohistological studies revealed proliferation of smooth muscle cells, which caused luminal stenosis of the VV. We also found decreased HemeB signals in the aortic wall of the sac as compared with those in the aortic wall of the neck region in AAA. The stenosis of adventitial VV in the AAA sac and the malperfusion of the aortic wall observed in the present study are new aspects of AAA pathology that are expected to enhance our understanding of this disease.

## Introduction

Abdominal aortic aneurysm (AAA) involves the progressive dilatation of the abdominal aorta as a consequence of degeneration. Currently,surgical repair is the only available method of treatment [1] since lack of knowledge regarding the pathogenesis of AAA has hindered the development of suitable medical treatments.

One of the proposed mechanisms of AAA development/rupture is hypoxia-mediated weakening of the wall [2], [3]. The aortic wall is normally maintained by direct perfusion from the vessel lumen or perfusion via the adventitial vaso vasorum (VV). The presence of an intraluminal thrombus (ILT) is thought to prevent the luminal perfusion of oxygen to the aortic wall, and this could cause tissue hypoxia. The role played byVV in the perfusion of the aortic wall in AAA remains unknown. The VV delivers nutrients and oxygen to the arterial wall and removes waste products produced in the wall [4]. Interestingly, the distribution of the VV in the abdominal aorta is known to be reduced in the infrarenal abdominal aorta as compared with that in the thoracic aorta [5].

We hypothesized that damage to the VV may be associated with disturbances in the delivery of nutrients and oxygen to the aortic wall, and thus may play an important role in the pathogenesis of AAA. In this study, we therefore examined the changes occurring in the adventitial VV in patients with AAA morphological analysis of VV, in fresh surgical samplesfrom patients undergoingopen repair of AAA. We further assessed the distribution of lipid molecules in the VV wall using matrix-assisted laser desorption/ionization imaging mass spectrometry (MALDI-IMS), to profile discrete cellular regions and obtain region-specific images, providing information on the relative abundance and spatial distribution of proteins, peptides, lipids, [6] and drugs [7].

## Materials and Methods

### Sample Collection

**Figure 1:**
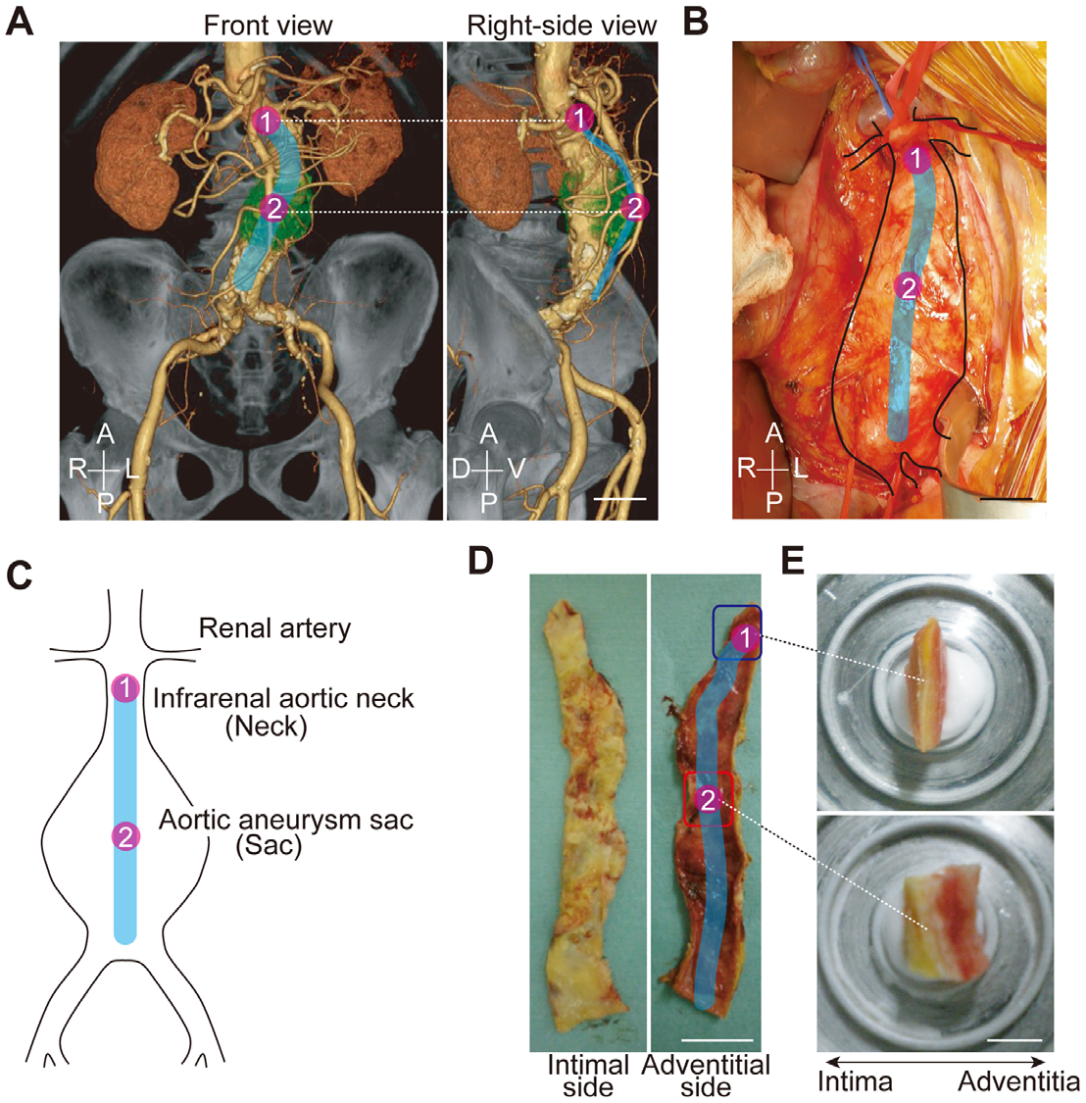
Sample harvesting during open repair of AAA. (A) Preoperative contrast-enhanced 3D-MDCT images of a patient (Table 1) with an AAA. A, anterior; D, dorsal; L, left; P, posterior; R, right; V, ventral. Scale bar = 2.0 cm. (B) Intraoperative view. Scale bar = 2.0 cm. (C) Schema of the AAA tissue. (D) Isolated tissue. Scale bar = 1.0 cm. (E) Frozen tissue before cross sectioning. Scale bar = 1.0 cm.

All procedures used in this study were approved by the Ethics Committee of Clinical Research of the Hamamatsu University School of Medicine, and with written consent was obtained from each patient. We enrolled 30 patients who underwent elective open surgery for repair of infrarenal AAAs at the Division of Vascular Surgery, Hamamatsu University School of Medicine, between April 2008 and April 2011. Aortic tissue samples were dissected during surgery abased on preoperative three-dimensional multi-detector computed tomography (3D-MDCT) imaging of the AAA being excised from the patient (Fig. 1A–C). Longitudinal tissue strips were selected from the infrarenal aortic neck (non-dilated normal aorta). Similarstrips extendinginto the region of maximal aneurysmal dilation were also obtained (Fig. 1D and 1E).

Eleven aorta samples obtained at autopsy were used as controls. The mid-portion of the abdominal aorta between the renal artery and the bifurcation was resected and collected from routine autopsies in the Department of Pathology, Hamamatsu University Hospital.

### Immunofluorescence Staining

Three tissue sections (8-μm thick) from the neck and sac of each AAA sample were obtained. The tissue sections were fixed with 4% paraformaldehyde in phosphate-buffered saline (pH 7.4) for 10 min at room temperature. The histological results from the VVs were assessed after staining using the following: rabbit anti-alpha smooth muscle actin (1∶100; Thermo Scientific, Waltham, MA, USA) or rabbit anti-Ki-67 (Ki-67) (1∶100; Abcam, Cambridge, MA, USA), mouse anti-Calprotectin-Monocyte/Macrophage (1∶100; Thermo Scientific), mouse anti-CD3e (1∶100; Thermo Scientific), goat anti-CD20 (1∶100; Santa Cruz Biotechnology, Inc., Santa Clara, CA, USA), rabbit anti-MMP-2 (1∶100; Thermo Scientific), mouse anti-MMP-9 (1∶100; Daiichi Fine Chemical Co., Ltd., Tokyo, Japan), goat anti-cathepsin S (1∶100; Santa Cruz Biotechnology, Inc.), and mouse anti- hypoxic inducible factor-1α (HIF-1α) (1∶200; Novus Biological, LLC, CO, USA).

The lumen and medial areas of the VV were measured in each section. The luminal area was defined as the area enclosed by the intima, while the intima-medial area was defined as the area enclosed between the external elastic laminae and the lumen. The average values of these areas in the adventitial VV from the neck and sac of AAA samples were compared. These areas were also measured in the adventitial VV of the control autopsy samples, specifically from the mid-portion of the infrarenal aorta.

### Imaging Mass Spectrometry

Matrix-assisted laser desorption (MALDI)/ionization mass spectrometry (IMS) was performed on the freshly harvested specimens from AAA patients. Three sections in the neck and sac of each AAA sample were obtained. Samples were prepared as described previously [6]. MALDI/IMS was performed using a time of flight (TOF) instrument (Ultraflex II, Bruker Daltonics Inc., Billerica, MA, USA) equipped with a 355-nm Nd:YAG laser at a repetition rate of 200 Hz. Data were acquired with a step size of 100 μm in the positive ion mode (reflector mode). A total of 500 μL of 2,5-dihydroxybenzoic acid (2, 5-DHB) solution in methanol/water (7/3, v/v) was used as the matrix. After analysis by IMS, the sections were subjected to hematoxylin-eosin (HE) staining.

### Identification of Biomolecules

To identify the peaks, MS/MS was performed using a MALDI-quadrupole ion trap (QIT)-TOF mass spectrometer (AXIMA-QIT; Shimadzu, Kyoto, Japan) [8]. The specific fragment patterns of HemeB, phosphatidylcholines (PCs), and cholesteryl esters (CEs) were annotated according to previous reports [8], [9].

### Statistical Analysis

The results were analyzed using StatView software (version 5.0; SAS Institute, Cary, NC, USA). All data were expressed as mean values ± SD. Statistical analysis was performed using analysis of variance for comparisons among the 3 groups (control, and neck and sac regions). Post-hoc comparison was performed using Tukey’s test. Comparisons between the neck and the sac regions were evaluated using the paired t test.

## Results

The clinical characteristics of the AAA patients are shown in Table 1. Eleven sex- and age-matched autopsy cases served as controls: their ages ranged from 54 to 89 years (mean age 71.2±9.7 years, all men). Of the control cases, 7 patients had died of cancer; 2, of renal failure; 1, of cerebral stroke; and 1, of cardiac infarction.

**Table 1:** Demographic and clinical data of the AAA patients.

Sex (n) (male/female)	26/4
Age	70.2±9.0
Height (m)	1.6±0.1
Weight (kg)	58.0±10.8
BMI (kg/m^2^)	21.9±2.4
Aortic diameter (mm)	
Neck	21.3±2.2
Sac	54.6±12.2
Serum TC (mg/dL)	192.3±30.8
HbA1c (%)	5.5±0.7
CRP (<0.3) (mg/dL)	1.0±2.2
Ever smoked (n)	30

Values are presented as mean ± SD unless stated otherwise.

Normal ranges: TC 130–240 mg/dL; HbA1c 4.3–5.8%; CRP 4.3–5.8 mg/dL.

AAA, abdominal aortic aneurysm; BMI, body mass index; TC, total cholesterol.

**Figure 2:**
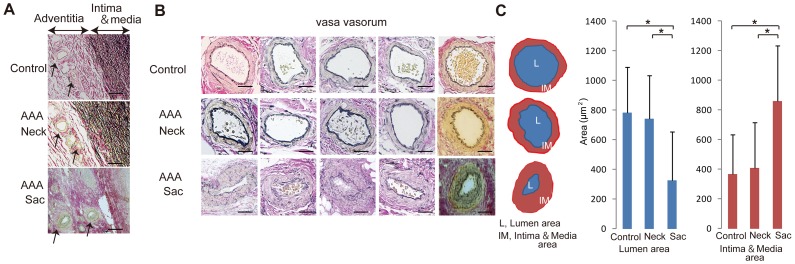
Adventitial vasa vasorum (VV) and its luminal stenosis. (A) Representative adventitial VV with Elastica Van Gieson (EVG) staining. Patent VV in the control and abdominal aortic aneurysm (AAA) neck, andstenotic VV in the AAA sac. Scale bar = 200 μm. (B) Comparison of the luminal and intima–medial areas among the control, neck, and sac adventitial VV. The luminal area was defined as the area enclosed by the intima, while the intima–medial area was defined as the area enclosed between the external elastic laminae and the lumen. Scale bar = 100 μm. (C) Data were obtained from 7 patients and 5 autopsied cases (controls). *P<0.001 indicates a statistically significant difference.

**Figure 3:**
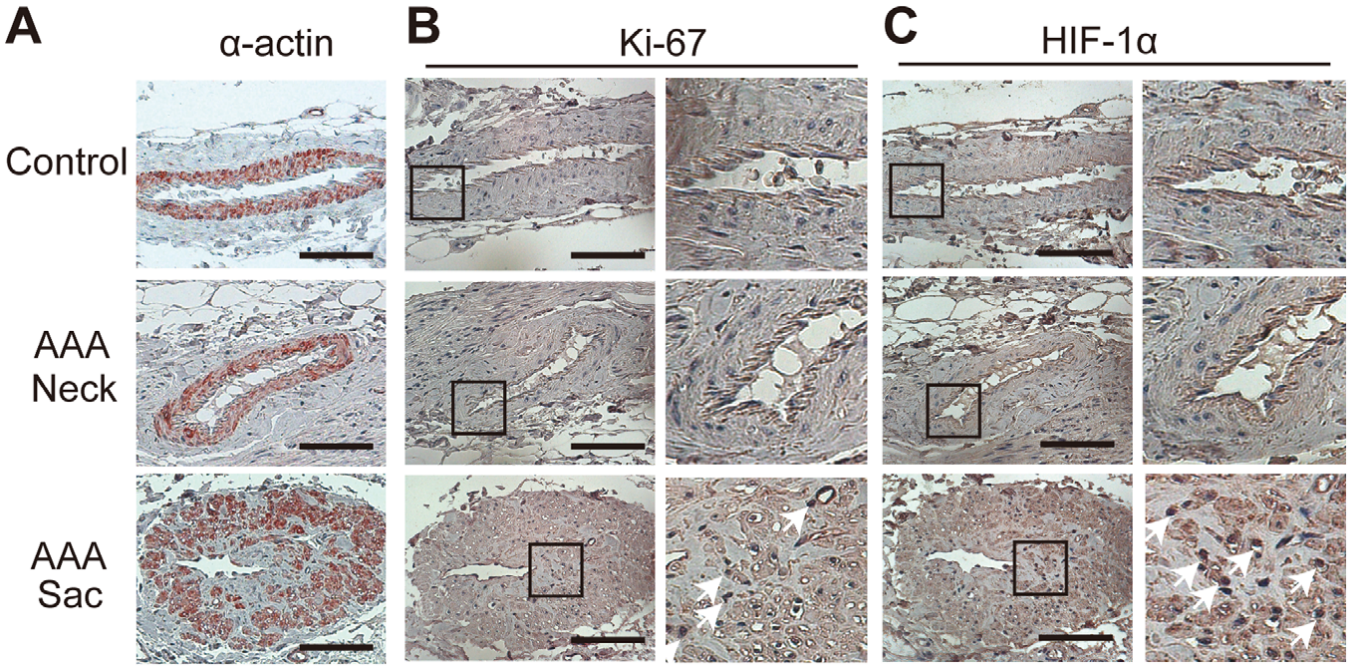
Immunohistological study of adventitial vasa vasorum (VV). (A) Immunostaining of SMCs in VV expressing alpha smooth muscle actin. Scale bar = 100 μm. (B) Immunostaining of cell proliferation marker (Ki-67) in VV. Scale bar = 20 μm. (C) Immunostaining of hypoxic inducible factor-1α (HIF-1α) in VV. Scale bar = 20 μm.

**Figure 4:**
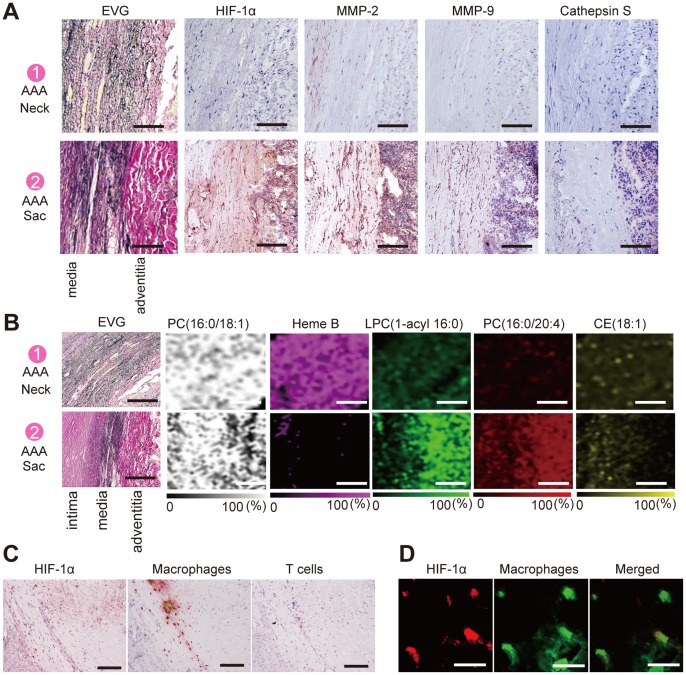
Distribution of lipid molecules and HemeB. (A) Elastica Van Gieson (EVG) staining and immunostaining of HIF-1α, MMP-2, MMP-9, and Cathepsin S in the aortic wall. Comparison of the distribution of these molecules in the aortic wall between the neck and sac regions. Brown color indicates the positive signals in all immunostaining pictures. Scale bar = 50 µm (from left to right). (B) Comparison of the distribution of Heme B, phosphatidylcholine (PC) (16∶0/18∶1), lyso-PC (LPC) (1-acyl 16∶0), PC(16∶0/20∶4), and cholesteryl ester (CE) (18∶1), in the aortic wall between the neck and sac regions, as analyzed by matrix-assisted laser desorption/ionization imaging mass spectrometry (MALDI-IMS). Scale bar = 100 μm. (C) HIF-1α, macrophages, and T cells in the AAA sac. Scale bar = 200 µm. (D) Immunofluorescence analysis of HIF-1α and macrophages in the AAA sac. Scale bar = 5 µm.

**Figure 5:**
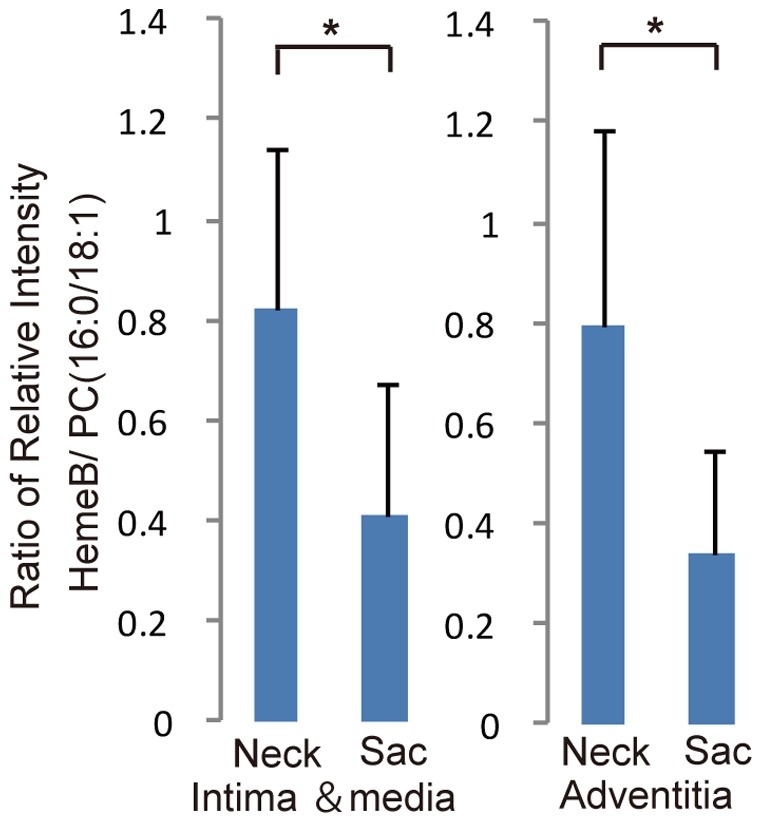
Comparison of HemeB signals. Comparisons of the relative intensity of Heme B/PC(16∶0/18∶1) in both intima–medial and adventitial regions in the AAA sac wall as compared with those in the neck wall. *P<0.001 indicates a statistically significant difference.

Histopathological analysis revealed marked intimal hyperplasia in the adventitial VV ofthe AAA sac with a compromised luminal area as compared with the VV in the non-dilated neck adventitia (Fig. 2A and 2B). Moreover, these changes in the VV were not observed in the control specimens. Due to the intimal hyperplasia, the luminal area of the VV in the sac adventitia was significantly smaller than that in the non-dilated neck adventitia or in the control specimens (Fig. 2C). Stenosis of the VV in the sac adventitia was associated with the proliferation of the intima-medial smooth muscle cells (SMCs) (Fig. 3A). Ki-67 staining was significantly more positive in the VV SMCs in the AAA sac than that observed in consecutive sections of the control and AAA neck VV (Fig. 3B). No atherosclerotic plaques were observed in the VV lumens in any of the specimens. Further, HIF-1α immunoreactivity was positive in the SMCs of the VV, suggesting that the VV wall was hypoxic (Fig. 3C). HIF-1α stained positive in all the layers of the AAA sac wall, which corresponded to the areas with weak Heme B signals (which is a specific marker for blood stock and may reflect the level of tissue blood flow) on MALDI-IMS (Fig. 4A and 4B). MALDI-IMS revealed that the relative intensity of Heme B (*m*/*z* 616) in the sac was significantly lower than that in the neck in both the intima and media as well as in the adventitia (Fig. 5). PC(16∶0/18∶1), the internal standard molecule, was detected ubiquitously both in the AAA neck and sac. Further, both MMP-2 and MMP-9 were strongly positive in the media and adventitia of the AAA sac wall, while cathepsin stained positively only in the adventitial side of the AAA sac wall. Co-immunostaining of macrophages/T-cells and HIF-1α identified that HIF-1α immunoreactivity was also positive in the macrophages in the AAA sac media and adventitia but negative in T-cells (Fig. 4C and 4D). MALDI-IMS further revealed that proinflammatory lipid molecules such as lyso-PC (LPC) (1-acyl 16∶0) and PC(16∶0/20∶4) were distributed in all the layers of the AAA sac; however, significantly greater accumulation of these molecules was observed in the adventitial side. In contrast, the distribution of cholesterol ester (CE) was not significantly different between the AAA neck and sac arterial walls.

**Figure 6:**
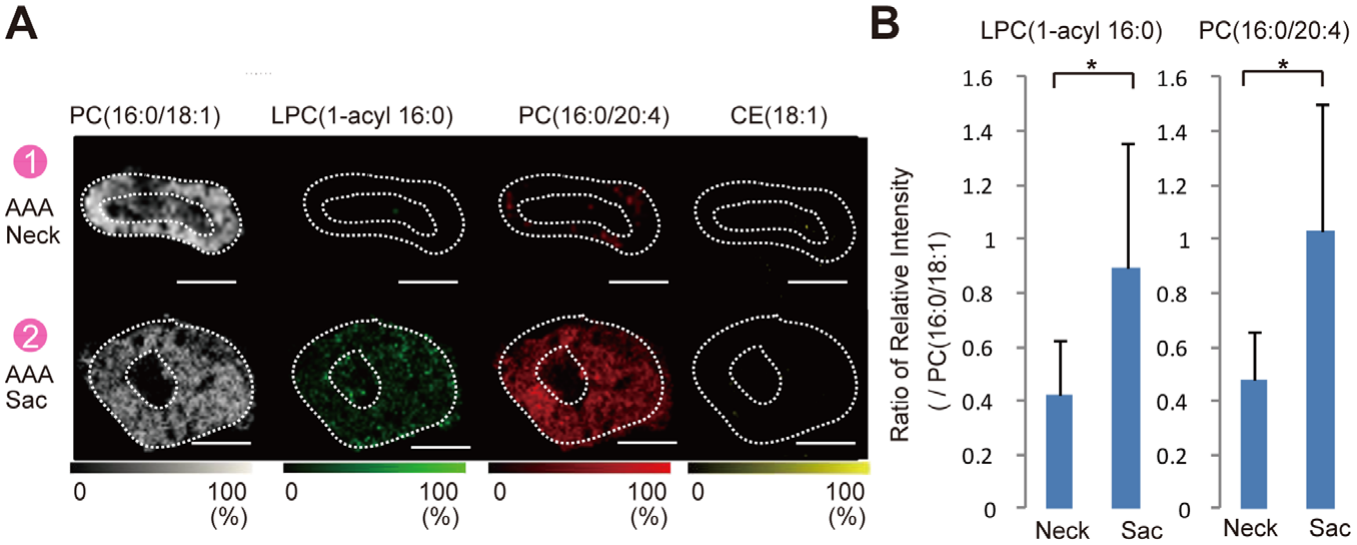
Distribution of lipid molecules in VV in high resolution. (A) MALDI-IMS. Comparison of the distribution of lipid molecules, phosphatidylcholine (PC) (16∶0/18∶1), lyso-PC (LPC) (1-acyl 16∶0), PC(16∶0/20∶4), and cholesteryl ester (CE) (18∶1), in the VV wall between the neck and sac regions analyzed by matrix-assisted laser desorption/ionization imaging mass spectrometry (MALDI-IMS). Scale bar = 20 μm. (B) Comparisons of the relative intensity of LPC(1-acyl 16∶0)/PC(16∶0/18∶1) and PC(16∶0/20∶4)/PC(16∶0/18∶1) in the VV wall between the neck and sac regions analyzed by MALDI-IMS. *P<0.001 indicates a statistically significant difference.

MALDI-IMS also showed characteristic localization of lyso-PC (LPC) (1-acyl 16∶0) and PC diacyl (16∶0/20∶4) in the sac VV as compared with the neck VV (Fig. 6A and 6B). CE(18∶1) was not detected either in the neck or sac VV, indicating no atherosclerotic lesions in the VV. PC diacyl (16∶0/18∶1), the internal standard molecule, was detected ubiquitously in both the neck and sac VV tissues.

## Discussion

The present study revealed significant stenosis of the adventitial VV in the arterial wall of the AAA sac, resulting from the proliferation of SMCs, which stained positive for HIF-1α. HIF-1α was also positive in the intima and medial layers, indicating hypoxia of the arterial wall. We further observed a decreased HemeB level in the sac wall, suggesting low perfusion of the sac. HemeB is the most abundant heme. It is synthesized and distributed in erythroid cells and hepatocytes, indicating that Heme B is a more specific marker for blood stock than formic acid or iron that can accurately reflect the tissue blood flow status [8] [10]. Therefore, in the present study, we assayed HemeB levels in order to examine the tissue blood flow.

The aortic wall is normally maintained by direct perfusion from the vessel lumen or perfusion via the adventitial VV. Inmost cases of AAA, large ILT occurs concurrently; therefore, oxygen delivery by direct perfusion from the lumen to the sac wall is expected to becompromised [11]. Among the 30 cases included in this study, 23 (>75%) cases showed the presence of a massive ILT, while no ILT was noted in 7 cases. We compared the two groups with/without ILT to assess whether the presence of ILT influenced adventitial VV arteriosclerosis or the expression of other molecules such as HIF-1α, Heme B, MMP-9, etc. However, no differences were observed between these two groups, indicating that changes in adventitial VV occurred irrespective of the presence of ILT (data not shown). Therefore, we consider that adventitial VV may play an independent role in the perfusion/oxygenation of the aortic wall, with VV stenosis contributing to the ischemia of the aortic wall itself. Given that the number of VV in the abdominal aorta is much less than that in the thoracic aorta, luminal stenosis of the sac VV could exacerbate tissue hypoxia in the abdominal aorta.

Pathologically, most aneurysms are characterized by upregulated proteolytic pathways within the medial layer of the arterial wall along with oxidative stress, adventitial inflammation, and loss of wall matrix [11], [12], [13]. Increased matrix metalloproteinase (MMP) activity has been implicated in aneurysm formation through elastin destruction and collagen degradation [14]. The concept that hypoxia can induce inflammation has gained general acceptance. The expression of HIF-1α is an adaptive response by the body against hypoxia, which in turn affects various inflammatory mediators [15]. MMP-2 one of these mediators, is activated under hypoxic conditions by a signaling cascade involving HIF-1α [16]. In our study, HIF-1α stained positive in both SMCs and macrophages in the AAA sac wall, indicating the occurrence of hypoxic conditions. Recently, Wan et al. reported that the accumulation of HIF-1α might be involved in the increased production of MMP-2 and MMP-9 in human monocytes [17]. Therefore, hypoxia (or HIF-1α) may accelerate MMP production from macrophages. Thus, intimal hyperplasia in the VV and the subsequent ischemia/hypoxia may be the major pathogenic mechanism involved in the development of AAA. However, the present study could not clarify whether VV stenosis occurred before degeneration and expansion of the aortic wall or resulted from changes occurring during the disease process. Further studies are required to clarify this issue.

VV stenosis occurs not because of atherosclerotic plaque formation but because of intimal hyperplasia. To the best of our knowledge, no epidemiological study has investigated the risk factors for VV stenosis. However, considering that VV are essentially endarteries, it is possible that aging, compression due to hypertension, and smoking could affect VV circulation; these factors are all known as important risk factors for AAA. Interestingly, the abdominal aorta appears to be particularly susceptible to the effects of smoking [18], [19].

Anatomically, VV originate from various arteries. The VV in the ascending aorta are derived from the coronary arteries and brachiocepharic artery. The VV in the descending thoracic aorta are derived from the intercostal arteries, while the VV in the abdominal aorta are derived from the lumbar and mesenteric arteries [20]. In AAA, the presence of an ILT frequently occludes the orifices of lumbar arteries or the inferior mesenteric artery, which may decrease the blood flow to the VV. The reduced blood flow in the VV could then cause proliferation of SMCs due to low wall shear stress [21], [22]. Hypoxia may also stimulate the proliferation of vascular SMCs via an HIF-1α-mediated pathway [23]. Indeed, in the sac adventitial VV SMCs, Ki-67 staining was significantly more positive than that in neck and control VV SMCs, suggesting that sac VV SMCs might have a greater proliferative ability than control and AAA neck VV SMCs [24]. Furthermore, inflammation may provoke release of growth factors and cytokines such as PDGF, FGF, IGF, and IL-1, which further induce intimal hyperplasia [25], [26], [27], [28].Thus, reduced VV blood flow, and the subsequent tissue hypoxia and inflammation may promote the proliferation of SMCs and accelerate the progression of luminal stenosis of VV, which in turn exacerbates hypoxia in the aortic wall.

To gain further insight into the pathogenesis of intimal hyperplasia in the VV, we analyzed the distribution of lipid molecules using MALDI-IMS. At present, MALDI-IMS is the only method available for the detailed assessment of lipid molecules in tissues. This method distinguishes between different lipid molecular species by simultaneously determining the differences in the mass/charge ratios (*m/z*). MALDI-IMS revealed abnormal accumulation of the lipid molecules LPC(1-acyl 16∶0) and PC(16∶0/20∶4) in the AAA sac’s medial and adventitial layers, where Heme B distribution was also remarkably reduced. Both LPC(1-acyl 16∶0) and PC(16∶0/20∶4) were also observed to be accumulated in VV tissues in the AAA sac wall. Since MALDI-IMS can only be performed using fresh samples, we could not evaluate the control samples using this method. However, abnormal accumulation of the lipid molecules was not observed in the neck VV tissue but was observed in the sac VV tissue. We have previously reported the accumulation of both PC(16∶0/20∶4) and LPC(1-acyl 16∶0) in intimal hyperplasia of the femoral artery in patients with peripheral artery occlusive disease. [9] Although the range of biochemical effects of PC (16∶0/20∶4) is largely unknown, elevated levels of the molecule have been reported in mammalian tissues with chronic inflammation [29], [30].

The PC class of lipids is a major component of most intracellular membranes. It is composed of a choline head group, a phosphoglycerol backbone, and 2 acyl chains of various combinations, which generate various PC species. PC(16∶0/20∶4) is an arachidonic acid-containing PC, which is degraded to arachidonic acid and LPC(1-acyl 16∶0) by phospholipase A2 [31]. Arachidonic acid induces proinflammatory signaling and interacts with substances in the vascular wall leading to the accumulation of foam cells and development of intimal hyperplasia [32], [33]. In animal models of aneurysm, leukotrienes which are produced from arachidonic acid have been reported to weaken the adventitia of the aorta [34]. Therefore, accumulation of arachidonic acid-containing PC in the VV wall may be implicated in an ongoing arachidonic acid-related inflammatory cascade. Moreover, the other molecule detected by MALDI-IMS, i.e., LPC, is known to exert a variety of biological activities, such as activating T-lymphocytes and enhancing adhesion molecule expression in endothelial cells [35]. It also plays an important role in the mitogenic activity of oxidized low-density cholesterol in monocyte-derived macrophages [36]. Therefore, the accumulation of PC(16∶0/20∶4) and LPC(1-acyl 16∶0) in the AAA sac VV could be associated with tissue inflammation, eventually leading to VV intimal hyperplasia.

### Conclusion

In this study, we demonstrated stenosis of the adventitial VV in the AAA sac with intimal hyperplasia of the VV. Accumulation of abnormal lipid molecules in the sac VV tissues was also observed. Immunohistological and MALDI-IMS findings indicated that the aortic wall of the AAA sac was ischemic and hypoxic. Our findings validate the role of changes in the VV of the AAA sac and the subsequent hypoxia/ischemia of the aortic wall in the pathogenesis of AAA.
